# Durable response rate as an endpoint in cancer immunotherapy: insights from oncolytic virus clinical trials

**DOI:** 10.1186/s40425-017-0276-8

**Published:** 2017-09-19

**Authors:** Howard L. Kaufman, Robert H. I. Andtbacka, Frances A. Collichio, Michael Wolf, Zhongyun Zhao, Mark Shilkrut, Igor Puzanov, Merrick Ross

**Affiliations:** 1 Rutgers Cancer Institute of New Jersey, 195 Little Albany Street, New Brunswick, NJ 08901 USA; 2 Huntsman Cancer Institute, University of Utah, 1950 Circle of Hope Drive, Salt Lake City, UT 84112 USA; 3 The University of North Carolina Chapel Hill, 170 Manning Drive, Box 7305, Chapel Hill, NC 27599 USA; 4 Amgen Inc., One Amgen Center Drive, Thousand Oaks, CA 91320 USA; 5 Department of Medicine, Roswell Park Cancer Institute, Elm and Carlton Streets, Buffalo, NY 14263 USA; 6 MD Anderson Cancer Center, 1515 Holcombe Blvd, Houston, TX 77030 USA

**Keywords:** Melanoma, Talimogene laherparepvec, Durable response rate, Patient-reported outcomes

## Abstract

**Background:**

Traditional response criteria may be insufficient to characterize full clinical benefits of anticancer immunotherapies. Consequently, endpoints such as durable response rate (DRR; a continuous response [complete or partial objective response] beginning within 12 months of treatment and lasting ≥6 months) have been employed. There has not, however, been validation that DRR correlates with other more traditional endpoints of clinical benefit such as overall survival.

**Methods:**

We evaluated whether DRR was associated with clinically meaningful measures of benefit (eg, overall survival [OS], quality of life [QoL], or treatment-free interval [TFI]) in a phase 3 clinical trial of an oncolytic virus for melanoma treatment. To evaluate the association between DRR and OS and to mitigate lead time bias, landmark analyses were used. QoL was evaluated using the FACT-BRM questionnaire (comprising the FACT-BRM Physical, Social/Family, Emotional, and Functional well-being domains, the Additional Concerns, Physical and Mental treatment-specific subscales, and the Trial Outcome Index [TOI]). TFI was defined as time from the last study therapy dose to first subsequent therapy dose (including any systemic anticancer therapy for melanoma after study therapy discontinuation).

**Results:**

Four hundred thirty-six patients were included in the intent-to-treat population. Achieving DR was associated with a statistically significant improvement in OS in a landmark analysis at 9 months (HR = 0.07; *P* = 0.0003), 12 months (HR = 0.05, *P* < 0.0001), and 18 months (HR = 0.11; *P* = 0.0002) that persisted after adjusting for disease stage and line of therapy. Achieving a DR was associated with a longer median TFI (HR = 0.33; *P* = 0.0007) and a higher TOI improvement rate (58.1% versus 30.0%; *P* = 0.025).

**Conclusions:**

Achieving a DR was associated with clinical benefits such as improved OS and QoL and prolonged TFI, thus supporting the usefulness of DR as a meaningful immunotherapy clinical trial endpoint.

**Trial registration:**

ClinicalTrials.gov identifier, NCT00769704 (https://clinicaltrials.gov/ct2/show/NCT00769704) October 7, 2008

**Electronic supplementary material:**

The online version of this article (doi:10.1186/s40425-017-0276-8) contains supplementary material, which is available to authorized users.

## Background

A growing body of clinical evidence indicates that traditional response criteria may not be sufficient to completely characterize the full spectrum of activities of current anticancer immunotherapies and/or define the clinical benefit associated with their use. Although a number of immunotherapies have recently been approved based, in part, upon tumor responses evaluated by traditional response criteria (eg, Response Evaluation Criteria in Solid Tumors [RECIST] [1, 2] or World Health Organization [WHO] criteria for response [3]), these criteria may not accurately capture the full benefits of immunotherapy-based drugs or regimens. In particular, these measures of response lack a durability dimension, and thus therapeutic benefit may be overestimated. Conversely, because responses may occur after an initial increase in tumor burden or appearance of new tumor lesions characterized as progressive disease by WHO criteria or RECIST [4–11], therapeutic benefit may be underestimated. Consequently, allowing for disease progression before response is critical for evaluation of immunotherapies because initial tumor growth may not always be indicative of treatment failure [4, 11].

Assuming response criteria that allow for progression prior to response are used, a conventional analysis is to evaluate duration of response for responders; however, no prospective criteria are designated for interpreting the clinical relevance of this information at the patient level or for treatment effect in a controlled study. To this end, the endpoint of durable response rate (DRR) has been proposed, which includes standard response criteria and a prospective durability dimension. Although DRR has not been validated as a surrogate endpoint for clinical benefit, anticancer agents have been approved based on a demonstrated ability to achieve retrospectively defined durable responses (DRs) [12–14]. With the allowance for progression prior to response, we hypothesized that DRR is an appropriate measure of the ability of immunotherapy anticancer agents to mediate a clinically meaningful, sustained reduction in tumor burden that may be associated with measures of clinical benefit such as prolonged overall survival (OS), improved quality of life (QoL), and freedom from successive anticancer therapies. This is especially important for immunotherapy-based regimens since the kinetics of response may be different when compared to cytotoxic chemotherapy or targeted therapy that mediate antitumor activity directly in tumor cells.

For this analysis, we used prospectively collected clinical data from the OPTiM trial, a randomized phase 3 study that compared intralesional talimogene laherparepvec, a genetically modified herpes simplex virus (HSV) type-1, designed to selectively replicate in tumors and produce human granulocyte macrophage-colony stimulating factor (GM-CSF) to subcutaneous GM-CSF, to evaluate whether DRR is associated with measures of clinical benefit, such as OS and QoL or treatment-free interval (TFI) after therapy. Talimogene laherparepvec is thought to mediate antitumor activity, at least in part, through activation of systemic immune responses and initial results from previous clinical trials demonstrated delayed kinetics of response similar to observations from T-cell checkpoint inhibitor trials [8, 15]. DRR, defined as a continuous response (complete response [CR]/partial response [PR]) beginning in the first 12 months of treatment and lasting 6 months or longer, was the primary endpoint in OPTiM. Although results from the OPTiM study have been reported previously [15], this analysis was undertaken to further validate the DRR endpoint by determining if DR is associated with other measures of clinical benefit. These data have implications for incorporating DRR as a regulatory endpoint in tumor immunotherapy clinical trial designs.

## Methods

### Study design

Study design and patient population for OPTiM have been previously described [15]. Briefly, eligible patients with histologically confirmed, surgically unresectable, stage IIIB/IIIC/IV melanoma that could undergo direct/ultrasound-guided injection of dermal, subcutaneous, or lymph node melanoma metastases were included. Patients were randomized 2:1 to receive intralesional talimogene laherparepvec or subcutaneous GM-CSF. The primary endpoint was DRR, defined as the rate of objective response (CR/PR) by modified WHO criteria lasting ≥6 months continuously and beginning within the first 12 months of initiating treatment where disease progression was allowed prior to onset of response. Key secondary endpoints included OS, best overall response, onset and duration of response, and time to treatment failure. Exploratory objectives included evaluation of patients’ QoL and analysis of the influence of achieving a response on OS.

### Analysis population

Data cutoff for DR and QoL analyses was December 2012, and data cutoff for OS and subsequent therapy analyses was February 2013. Data cutoff for duration of response per investigator was August 2014 (final analysis). A landmark approach was used to limit lead-time bias because the minimum time required to achieve a DR was 9 months (ie, 3 months to first tumor assessment plus minimum 6 months of response duration). Analyses excluded patients with a follow-up duration less than the landmark time.

### Assessments

Assessment of durable response in OPTiM has been described previously [15]. Briefly, visible/palpable lesions were assessed by clinical evaluation performed on day 1 of each cycle; computed tomography assessments of response were performed at baseline and every 12 weeks thereafter. Patients who had a best response of CR/PR per modified WHO criteria based on investigator assessment or were on therapy ≥9 months were evaluated for response by a blinded endpoint assessment committee (EAC) [15]. Patients were considered in response if there was ≥50% reduction in total tumor burden of all measurable lesions compared with baseline or, if any new tumors/lesions had appeared, compared with when they were first documented. Disease progression was considered a >25% increase in total tumor burden or appearance of new lesions, and clinically relevant disease progression was defined as disease progression associated with a decline in performance status and/or required alternative therapy. Treatment continued for 24 weeks, irrespective of progression. After 24 weeks, treatment continued until clinically relevant disease progression, intolerability, withdrawal of consent, complete remission, lack of response by 12 months, or disappearance of all injectable lesions (talimogene laherparepvec only) [15]. After 12 months, patients with stable/responding disease could continue treatment for 6 additional months.

Quality of life was assessed using a validated Functional Assessment of Cancer Therapy–Biologic Response Modifier (FACT-BRM) questionnaire, which comprises four domains (Physical, Social/Family, Emotional, and Functional well-being) and two treatment-specific subscales (Additional Concerns: Physical and Mental) [16]. The Trial Outcome Index (TOI) is a 27-item sum of the scores of the Physical and Functional well-being domains and the two treatment-specific subscales of FACT-BRM that has been shown to be a helpful single scale instrument for assessment of QoL in clinical trials [17, 18]. Changes in QoL were evaluated using the TOI as well as the FACT-BRM Total, FACT-BRM subdomains, and treatment-specific subscales. The questionnaire was completed before study procedures were conducted on day 1 of each treatment cycle (5 weeks for the first cycle and 4 weeks for subsequent treatment cycles) until the end of treatment and at the end-of-treatment visit (30 days after last treatment). In this analysis, a clinically meaningful improvement in QoL was defined as a ≥5-point increase from baseline lasting ≥28 days for the TOI and FACT-BRM Total and a ≥2-point increase for all other domains and Additional Concerns [19]. A minimum clinically important difference for TOI of 5 to 7 points has been established for group changes.

Melanoma treatments administered after discontinuation of talimogene laherparepvec/GM-CSF were recorded during the survival follow-up. Subsequent therapy was defined as any systemic anticancer therapy for melanoma after discontinuation of study therapy and was categorized as chemotherapy/targeted agents or immunotherapy.

### Statistical analysis

The primary analysis was planned when no further patients had the possibility of meeting the criteria for durable response; the final OS analysis was planned for after all patients had reached 18 months from their first dose of treatment. To mitigate potential for lead-time bias (ie, patients who live long enough to have a DR tend to have longer survival, irrespective of whether durable response prolongs survival), OS for patients who had achieved a DR and were alive was compared with OS for patients who had not achieved a DR and were alive in a landmark analysis at 9, 12, and 18 months after randomization [20]. In this analysis, OS was calculated from a landmark time to death; patients who died or who were censored before the landmark time were excluded from the analysis. Because disease stage and line of therapy were found to be associated with DR and OS in multivariate analyses and because the talimogene laherparepvec treatment effect on DRR and OS was pronounced in patients with stage IIIB/IIIC/IVM1a melanoma and treatment-naive patients in exploratory subgroup analyses of OPTiM, Cox model hazard ratio estimates and log-rank tests in landmark analysis were stratified by these factors, accounting for observed baseline imbalances (ie, disease stages IIIB/IIIC/IVM1a versus IVM1b/IVM1c, first-line versus second-line or later therapy).

To further evaluate any potential association between achievement of DR and OS, the time when DR was achieved was also evaluated in a Cox proportional hazards model as a time-dependent covariate for all randomized patients. Treatment-free interval (defined as time from the last dose of study therapy to first dose of subsequent therapy or censoring in the absence thereof at end of follow-up) was estimated using the Kaplan-Meier method. As with OS, the association between TFI and achieving a DR was evaluated in a landmark analysis using a Cox proportional hazards model and log-rank test. The TOI analysis set included patients with a baseline score (defined as the maximum possible score—5-point absolute increase criterion for improvement), ≥1 post-baseline non-missing score, and tumor assessments for ≥9 months of follow-up. Associations between FACT-BRM improvement and achievement of DR were evaluated using an odds ratio stratified by disease stage and line of therapy in a landmark analysis at 9 months after randomization. Odds ratios for those who achieved a DR versus those who did not and *P* values for TOI improvement were obtained by fitting an exact logistic regression model using DR status as a predictor and TOI improvement as response. Statistical significance was evaluated at a nominal two-sided 0.05 significance level without multiplicity assessment.

## Results

### Patients

In total, 436 patients were included in the intent-to-treat population (295 in the talimogene laherparepvec arm and 141 in the GM-CSF arm). Baseline demographic/clinical characteristics for the study population are summarized in Table 1 and have been reported previously by arm [15]. At the primary analysis, 86 patients in the intent-to-treat population achieved an OR per EAC and, of these, the OR duration did not qualify for a DR for 35 patients but it did for 51 (talimogene laherparepvec, *n* = 48; GM-CSF, *n* = 3), 23 of whom had progression before response (all in the talimogene laherparepvec arm). Among the 86 patients with an OR, a similar proportion of DR versus non-DR patients had early stage disease (82% versus 74%) and received first-line therapy (65% versus 63%). At the final analysis, 59 patients achieved a DR per investigator (talimogene laherparepvec, *n* = 57; GM-CSF, *n* = 2; Additional file 1: Figure S1), indicating that responses continued to evolve over time.

**Table 1:** Baseline Demographics and Clinical Characteristics for Patients Treated with Talimogene Laherparepvec and GM-CSF

Characteristics	All Patients^a^(*N* = 436)	Patients with Durable Response (*N* = 51)
Median age (range), years	63 (22–94)	70 (36–91)
Sex, n (%)		
Men	250 (57)	31 (61)
Women	186 (43)	20 (39)
Disease substage, n (%)^b^		
IIIB	34 (8)	10 (20)
IIIC	97 (22)	19 (37)
IVM1a	118 (27)	13 (26)
IVM1b	90 (21)	3 (6)
IVM1c	96 (22)	6 (12)
Missing	1 (<1)	0 (0)
LDH, n (%)^a^		
≤ ULN	390 (89)	47 (92)
> ULN	20 (5)	0 (0)
Line of therapy, n (%)		
First-line	203 (47)	33 (65)
Second or greater	233 (53)	18 (35)
ECOG performance status, n (%)^a^		
0	306 (70)	41 (80)
1	114 (26)	10 (20)
Missing	16 (4)	0 (0)
HSV serostatus, n (%)^a^		
Positive	142 (33)	34 (67)
Negative	253 (58)	13 (26)
Unknown/missing	41 (9)	4 (8)
*BRAF* status, n (%)		
Mutation	69 (16)	5 (10)
Wild-type	68 (16)	6 (12)
Unknown/missing	299 (68)	40 (78)
Treatment assignment, n (%)		
Talimogene laherparepvec^c^	295 (68)	48 (94)
GM-CSF^d^	141 (32)	3 (6)

*DRR* durable response rate, *ECOG* Eastern Cooperative Oncology Group, *GM-CSF* granulocyte macrophage-colony stimulating factor, *HSV* herpes simplex virus, *LDH* lactate dehydrogenase, *ULN* upper limit of normal

^a^Intent-to-treat population

^b^Includes one patient with unknown disease stage

^c^4 patients in the talimogene laherparepvec arm were not treated with talimogene laherparepvec

^d^11 patients in the GM-CSF arm were not treated with GM-CSF

### Association between durable response and overall survival

**Fig. 1:**
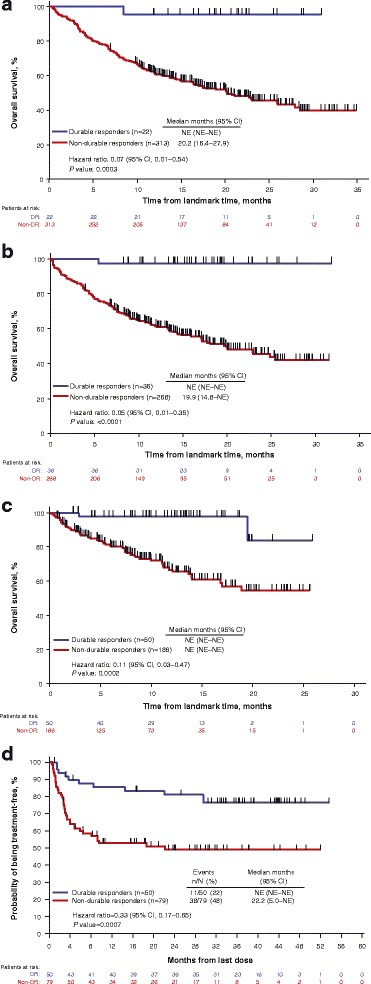
Kaplan-Meier plots of overall survival in patients who achieved a durable response versus patients who did not achieve a durable response prior to landmark times of 9 months (**a**), 12 months (**b**), and 18 months from randomization (**c**), and treatment-free survival in patients who achieved a durable response versus patients who did not achieve a durable response (**d**). For landmark analyses, OS was calculated from the landmark time (9, 12, or 18 months after randomization) to death. TFI analysis was limited to treated patients with tumor assessments ≥9 months. Unadjusted hazard ratios (HR) and log-rank *P* values are shown. *DR* durable response, *NE* not evaluable, *OS* overall survival, *TFI* treatment-free interval

Achieving a DR was associated with a statistically significant improvement in OS in a landmark analysis at 9 months (HR = 0.07; 95% CI, 0.01–0.48; *P* = 0.0003; Fig. 1a), at 12 months (HR = 0.05, 95% CI, 0.01–0.33; *P* < 0.0001; Fig. 1b), and at 18 months (HR = 0.11; 95% CI, 0.03–0.44; *P* = 0.0002; Fig. 1c). Because comparisons of OS and DR may be confounded (eg, by lead-time bias), we employed analytical techniques to mitigate against such potential biases. The association between DR and OS remained after adjusting for potentially confounding imbalances in disease stage and line of therapy (both were shown to be predictive for treatment effect of talimogene laherparepvec in post hoc exploratory analyses [15]) between those who achieved a DR and those who did not (9 months, HR = 0.07; 95% CI, 0.01–0.54; 12 months, HR = 0.05; 95% CI, 0.01–0.035; 18 months, HR = 0.11, [95% CI, 0.03–0.47]; Table 2). Consistent results were obtained when achievement of DR was evaluated in a Cox proportional hazards model as a time-dependent covariate among all randomized patients (HR = 0.08; 95% CI, 0.03–0.26).

**Table 2:** Incidence of DR at Each Landmark Time Assessment in OPTiM Patients Treated with Talimogene Laherparepvec and GM-CSF^a^

Landmark Time, Months	Patients Alive, n	DR Achieved, n	HR for OS in Patients with DR Versus Those Without, HR (95% CI)	Adjusted HR for OS in Patients with DR Versus Those Without HR (95% CI)^b^
9	335	22	0.07 (0.01–0.48)	0.07 (0.01–0.54)
12	304	36	0.05 (0.01–0.33)	0.05 (0.01–0.35)
18	236	50	0.11 (0.03–0.44)	0.11 (0.03–0.47)

*DR* durable response, *GM-CSF* granulocyte macrophage-colony stimulating factor, *HR* hazard ratio, *ITT* intent-to-treat, *OS* overall survival

^a^Analysis was performed in ITT population of OPTiM

^b^Adjusted for disease stage (stage IIIB, IIIC, IVM1a versus stage IVM1b, IVM1c) and line of therapy (first-line versus second-line)

### Durable response and treatment-free interval

Of the 436 patients included in the intent-to-treat population, 129 received study therapy and had tumor assessments at ≥9 months of follow-up and were included in the TFI analysis; of these, 50 patients achieved a DR per the EAC (talimogene laherparepvec, *n* = 47; GM-CSF, *n* = 3) and 79 patients did not (talimogene laherparepvec, *n* = 65; GM-CSF, *n* = 14). Of the 50 patients with a DR, 11 (22%) were treated with subsequent systemic anti-melanoma therapy after the end of their assigned treatment with GM-CSF/talimogene laherparepvec (talimogene laherparepvec, *n* = 9; GM-CSF, *n* = 2). Of 79 patients without a DR, 38 (48%) were treated with subsequent systemic anti-melanoma therapy (talimogene laherparepvec, *n* = 28; GM-CSF, *n* = 10).

Kaplan-Meier plots of freedom from subsequent systemic anti-melanoma therapy in patients who achieved a DR versus patients who did not achieve a DR with tumor assessments at ≥9 months, including hazard ratio (HR) and log-rank *P* value, are shown in Fig. 1. DR was associated with a significantly longer median TFI (HR = 0.33; 95% CI, 0.17–0.65; *P* = 0.0007; Fig. 1d). Achieving a DR was also associated with a 27.5% (95% CI, 10.6–44.4) reduced risk of initiating subsequent systemic therapy at 36 months (DR, 76.5% [95% CI, 61.4–86.2]; no DR, 49.0% [95% CI, 36.9–60.0]).

**Fig. 2:**
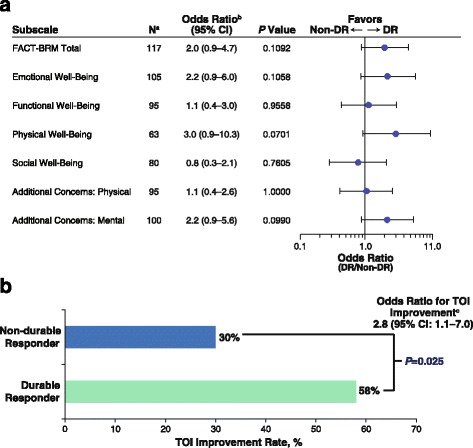
Association of durable response with (**a**) FACT-BRM domain and subscale improvement and (**b**) TOI Improvement. ^a^Analysis was limited to patients with tumor assessments ≥9 months evaluable for improvement. ^b^Odds ratio stratified by disease stage and lines of therapy. ^c^Only patients followed up for ≥9 months included. *DR* durable response, *FACT-BRM* Functional Assessment of Cancer Therapy Biologic Response Modifier, *HR* hazard ratio, *TOI* Trial Outcome Index

### Quality of life

Of the 129 patients with tumor assessments at ≥9 months of follow-up, 117 had QoL data and 103 had data for the TOI analysis. Overall, achieving a DR was associated with improved scores in the TOI, the FACT-BRM Total, and all FACT-BRM domains and treatment-specific subscales, except for social well-being (Fig. 2a).

Achievement of a DR was associated with a higher TOI improvement rate. Among patients who achieved a DR, 58.1% (25/43) had a clinically meaningful TOI improvement, compared with 30.0% (18/60) of patients who did not achieve a DR (*P* = 0.025; Fig. 2b). In a landmark analysis limited to patients with ≥9 months of follow-up for tumor response assessment, the odds ratio for TOI improvement was 2.8 (95% CI, 1.1–7.0; Table 3). A sensitivity analysis evaluated whether the association was retained when TOI improvement required a larger absolute increase from baseline and/or longer improvement duration. Although nominal 0.05 significance was not achieved in all cases, it was achieved for a magnitude up to 9 points or a duration up to 4 cycles. In addition, with one exception, odds ratios were greater than 1 for all magnitude and duration combinations (Additional file 1: Table S1).

**Table 3:** TOI Association with Achieving a DR (per Endpoint Assessment Committee)^a^

Improvement Magnitude, Points	Improvement Duration, Cycles	TOI Improvement Rate, Odds Ratio^b^ (95% CI)
5	1	2.8 (1.1–7.0)
	3	2.6 (1.0–6.9)
6	1	3.0 (1.2–7.8)
	3	2.9 (1.1–8.1)
	4	3.1 (1.1–9.1)
7	1	3.1 (1.2–8.4)
	2	2.8 (1.1–7.6)
	3	3.0 (1.0–9.4)
8	1	3.1 (1.1–8.5)
	2	2.8 (1.0–7.8)

*DR* durable response, *TOI* Trial Outcome Index

^a^Intent-to-treat landmark analysis patients with ≥9 months’ follow-up evaluable for TOI improvement were included; only odds ratio of TOI improvement rates with corresponding *P* values <0.05 are shown. Full results are available in Additional file 1: Material

^b^Odds ratio (DR/non-DR) stratified by disease stage (IIIB/IIIC/IVM1a versus IVM1b/IVM1c) and line of therapy (first-line versus second-line or later therapy)

## Discussion

Data from this analysis of outcomes in the phase 3 OPTiM study indicate that achievement of a DR in patients with unresectable regionally and distantly advanced melanoma was associated with several independent clinical benefits, providing further support for DR as a useful endpoint in patients with melanoma. Achievement of a DR was associated with improved OS in landmark analyses at 9, 12, and 18 months, lower risk of subsequent systemic therapy use at 36 months, and improved QoL at ≥9 months of follow-up. The risk of death was decreased by approximately 90% for patients with a DR. Similar results were obtained in analyses adjusted for imbalances in baseline demographic/clinical characteristics and in a Cox proportional hazards model that evaluated achievement of DR as a time-dependent covariate. Furthermore, patients with a DR have an approximately two-thirds reduction in the risk of requiring a new therapy and were approximately 3 times more likely to have a clinically meaningful improvement in QoL. The association with improvement in QoL is particularly notable in the melanoma setting where skin lesions may be regarded as disfiguring by patients. Taken together, this evidence indicates that DR is an efficacy endpoint that is associated with other favorable clinical outcomes.

Since the proposed mechanism of action of talimogene laherparepvec includes release of tumor-associated antigens and cell- and damage-associated molecular pattern molecules, and local expression of GM-CSF, talimogene laherparepvec is likely able to recruit and expand tumor associated antigen-reactive T cells [21]. This process may take time, and lesions may continue to grow before regression. Furthermore, this process may cause a local inflammatory-like response in lesions, resulting in a period of “pseudo-progression” before response [15, 22]; thus, alternative measures of clinical response may be needed. These observations of progression before response are consistent with the immune-mediated antitumor activity associated with the talimogene laherparepvec mechanism of action, and have been reported with other immunotherapy agents such as ipilimumab [23]. With the allowance for progression before response, our results demonstrate the utility of DR as an appropriate endpoint for oncolytic virus clinical trials and for trials involving combination regimens of immunotherapeutic agents. An element of DR that may be a key to its clinical value is that it incorporates a time component: responses must be maintained for ≥6 months continuously and begun within the first 12 months of initiating treatment. In contrast, the WHO criteria and RECIST do not make allowance for progression before response or define a meaningful minimum duration of response. In a randomized clinical trial of ipilimumab in melanoma, a significant improvement in OS was seen; however, treatment had no impact on progression-free survival (PFS) [10]. These results highlight the inherent problems in using PFS using conventional response criteria as a primary endpoint in immunotherapy clinical trials, and the need for identification of alternative endpoints to accurately assess immunotherapy drugs in early phase clinical development where OS may not be a feasible endpoint. Our data support the use of DRR as a flexible and appropriate surrogate of OS and other measures of clinical benefit.

Other attempts to define alternative clinical trial endpoints for immunotherapy agents include modifications of RECIST (such as immune-related response criteria; irRC [4]), which are being used to evaluate tumor responses in clinical trials across a variety of tumor types [24]. A benefit to the use of irRC is that progressive disease prior to treatment response is permitted, consistent with the criteria for DR in the OPTiM study. However, like the WHO criteria and RECIST, irRC does not define a meaningful duration for tumor response. Furthermore, endpoints such as objective response or progression-free survival based on irRC have not yet been validated as correlates of OS.

Unlike survival endpoints, response indicates a direct biological effect of a therapy on the tumor. Patients can survive for prolonged periods without treatment, but tumors rarely recede or disappear without intervention. To be considered a DR, however, the response must be maintained for a period of at least 6 months. This duration component may make achieving a DR a more descriptive endpoint than others such as objective response, which requires only response confirmation at least 4 weeks after the initial assessment of response. Six months was initially selected as a reasonable duration because most non-immunotherapy treatments rarely induce responses beyond 6 months in melanoma [25]. The expanded use of DRR for other cancers allows for the selection of the most appropriate duration based on the natural history of the disease and known impact of established therapeutic regimens. Thus, by altering the duration of response, DRR can be used in a more flexible manner for other cancers that may exhibit different natural history and/or have other standard treatments available that extend survival for variable times. The inclusion of the initiation at any time within 12 months of starting treatment allows capturing pseudoprogression, which has been reported in some immunotherapy trials, and permits continued treatment beyond clinically asymptomatic progression, as has been reported for irRC proposed for tumor immunotherapy studies [4]. It is also possible that a rate of durable stable disease might represent an informative endpoint; however, further evaluation would be required to validate such an endpoint.

This study had certain limitations. First, these analyses were exploratory and should therefore be interpreted with caution. Second, although we identified associations between DR and clinical outcomes, causative relationships could not be evaluated. For example, although our analysis indicated an association between DR and OS, it is possible that factors other than achievement of DR (eg, imbalances in disease stage and line of therapy, or subsequent therapy) might have influenced the observed outcome. Although endeavors were made to mitigate the potential for confounding by such factors, it is difficult to determine how successful they might have been. Additionally, the selection of 6 months as the minimum duration for achievement of a durable response was based on responses to cytotoxic chemotherapy agents; this time point was, to an extent, arbitrary. In the OPTiM study, some patients had DRs that lasted substantially longer than 6 months. It is unknown whether the associations found in this analysis would have been observed had the distribution of DR duration been closer to the 6-month minimum. Finally, it is possible that the associations seen were driven by the preferential occurrence of complete response among patients with DR (of any duration).

## Conclusions

Results from this analysis indicate that the achievement of a DR in patients with unresectable regionally and distantly advanced melanoma was associated with improved clinical benefit and QoL. Although causal relationships cannot be determined via these analyses, these findings support the utility of DR as a meaningful trial endpoint when assessing efficacy of immunotherapies for solid tumors. Further consideration of durable response rate as a clinical endpoint in cancer immunotherapy trials is also warranted and may allow for improved selection of promising agents for later phase clinical development.

## Additional file

Additional file 1:
**Table S1.** TOI Improvement Rate Association with Achievement of a DR (per EAC). **Figure S1.** Talimogene laherparepvec Duration of Response (per Investigator). As of the final analysis 93 of 295 patients (31.5%) randomized to talimogene laherparepvec had an overall response per investigator assessment (complete response, *n* = 50; partial response, *n* = 43) and 57 patients (19.3%) had a durable response. (ZIP 1510 kb)

## Abbreviations

CR: Complete response
DR: Durable response
DRR: Durable response rate
EAC: Endpoint assessment committee
FACT-BRM: Functional Assessment of Cancer Therapy-Biologic Response Modifier
GM-CSF: Granulocyte macrophage-colony stimulating factor
HR: Hazard ratio
HSV: Herpes simplex virus
irRC: Immune-related response criteria
OS: Overall survival
PFS: Progression-free survival
PR: Partial response
QoL: Quality of life
RECIST: Response Evaluation Criteria in Solid Tumors
TFI: Treatment-free interval
TOI: Trial outcome index
WHO: World Health Organization
