# Supplementary material for: Durable response rate as an endpoint in cancer immunotherapy: insights from oncolytic virus clinical trials
Source: J Immunother Cancer. 2017 Sep 19;5:72. doi: 10.1186/s40425-017-0276-8 (PMC5604502; doi:10.1186/s40425-017-0276-8)
Supplement: Additional file 1: — Table S1. TOI Improvement Rate Association with Achievement of a DR (per EAC). Figure S1. Talimogene laherparepvec Duration of Response (per Investigator). As of the final analysis 93 of 295 patients (31.5%) randomized to talimogene laherparepvec had an overall response per investigator assessment (complete response, n = 50; partial response, n = 43) and 57 patients (19.3%) had a durable response. (ZIP 1510 kb) [file 40425_2017_276_MOESM1_ESM.zip › Kaufman et al_supplement.docx]

# Durable Response Rate as an Endpoint in Cancer Immunotherapy: Insights from Oncolytic Virus Clinical Trials

Howard L. Kaufman,^1^ Robert H. I. Andtbacka,^2^ Frances A. Collichio,^3^ Michael Wolf,^4^ Zhongyun Zhao,^4^ Mark Shilkrut,^4^ Igor Puzanov,^5^ Merrick Ross^6^

^1^Rutgers Cancer Institute of New Jersey, New Brunswick, NJ; ^2^Huntsman Cancer Institute, University of Utah, Salt Lake City, UT; ^3^The University of North Carolina Chapel Hill, Chapel Hill, NC; ^4^Amgen Inc., Thousand Oaks, CA; ^5^Department of Medicine, Roswell Park Cancer Institute, Buffalo, NY; ^6^MD Anderson Cancer Center, Houston, TX

# SUPPLEMENTAL MATERIAL

# Supplementary Table 1. TOI Improvement Rate Association with Achievement of a DR (per EAC)

| **Magnitude (Points)** | **Duration (Cycles)** | **Overall n/N(%)** | **DR n/N (%)** | **Non-DR n/N (%)** | **Odds Ratio* (–)** | ***P*-value*** |
| --- | --- | --- | --- | --- | --- | --- |
| 5 | 1 | 43/103 (41.7) | 25/43 (58.1) | 18/60 (30.0) | 2.77 (1.12–7.02) | 0.03 |
|  | 2 | 38/103 (36.9) | 21/43 (48.8) | 17/60 (28.3) | 2.00 (0.81–5.03) | 0.15 |
|  | 3 | 33/103 (32.0) | 20/43 (46.5) | 13/60 (21.7) | 2.60 (1.01–6.93) | 0.048 |
|  | 4 | 29/103 (28.2) | 17/43 (39.5) | 12/60 (20.0) | 2.19 (0.83–5.91) | 0.12 |
|  | 5 | 27/103 (26.2) | 16/43 (37.2) | 11/60 (18.3) | 2.24 (0.83–6.23) | 0.12 |
|  | 6 | 26/103 (25.2) | 16/43 (37.2) | 10/60 (16.7) | 2.53 (0.92–7.20) | 0.08 |
| 6 | 1 | 39/99 (39.4) | 23/41 (56.1) | 16/58 (27.6) | 2.98 (1.18–7.78) | 0.02 |
|  | 2 | 33/99 (33.3) | 19/41 (46.3) | 14/58 (24.1) | 2.37 (0.91–6.25) | 0.08 |
|  | 3 | 29/99 (29.3) | 18/41 (43.9) | 11/58 (19.0) | 2.88 (1.06–8.07) | 0.04 |
|  | 4 | 25/99 (25.3) | 16/41 (39.0) | 9/58 (15.5) | 3.05 (1.08–9.12) | 0.03 |
|  | 5 | 23/99 (23.2) | 14/41 (34.1) | 9/58 (15.5) | 2.52 (0.87–7.66) | 0.10 |
|  | 6 | 22/99 (22.2) | 14/41 (34.1) | 8/58 (13.8) | 2.91 (0.98–9.18) | 0.06 |
| 7 | 1 | 31/96 (32.3) | 19/39 (48.7) | 12/57 (21.1) | 3.07 (1.16–8.40) | 0.02 |
|  | 2 | 30/96 (31.3) | 18/39 (46.2) | 12/57 (21.1) | 2.78 (1.05–7.60) | 0.04 |
|  | 3 | 22/96 (22.9) | 14/39 (35.9) | 8/57 (14.0) | 2.99 (1.01–9.40) | 0.048 |
|  | 4 | 20/96 (20.8) | 12/39 (30.8) | 8/57 (14.0) | 2.42 (0.79–7.75) | 0.13 |
|  | 5 | 18/96 (18.8) | 10/39 (25.6) | 8/57 (14.0) | 1.92 (0.59–6.39) | 0.33 |
|  | 6 | 17/96 (17.7) | 10/39 (25.6) | 7/57 (12.3) | 2.27 (0.68–7.94) | 0.21 |
| 8 | 1 | 30/93 (32.3) | 18/37 (48.6) | 12/56 (21.4) | 3.06 (1.14–8.50) | 0.02 |
|  | 2 | 27/93 (29.0) | 16/37 (43.2) | 11/56 (19.6) | 2.78 (1.02–7.83) | 0.046 |
|  | 3 | 20/93 (21.5) | 12/37 (32.4) | 8/56 (14.3) | 2.61 (0.85–8.43) | 0.10 |
|  | 4 | 18/93 (19.4) | 10/37 (27.0) | 8/56 (14.3) | 2.06 (0.64–6.88) | 0.27 |
|  | 5 | 18/93 (19.4) | 10/37 (27.0) | 8/56 (14.3) | 2.06 (0.64–6.88) | 0.27 |
|  | 6 | 15/93 (16.1) | 9/37 (24.3) | 6/56 (10.7) | 2.55 (0.72–9.75) | 0.17 |
| 9 | 1 | 27/85 (31.8) | 16/34 (47.1) | 11/51 (21.6) | 2.80 (1.00–8.12) | 0.050 |
|  | 2 | 21/85 (24.7) | 10/34 (29.4) | 11/51 (21.6) | 1.40 (0.46–4.28) | 0.67 |
|  | 3 | 16/85 (18.8) | 8/34 (23.5) | 8/51 (15.7) | 1.55 (0.44–5.45) | 0.61 |
|  | 4 | 14/85 (16.5) | 7/34 (20.6) | 7/51 (13.7) | 1.55 (0.41–5.88) | 0.65 |
|  | 5 | 13/85 (15.3) | 6/34 (17.6) | 7/51 (13.7) | 1.29 (0.32–5.08) | 0.90 |
|  | 6 | 12/85 (14.1) | 6/34 (17.6) | 6/51 (11.8) | 1.56 (0.37–6.60) | 0.69 |
| 10 | 1 | 23/81 (28.4) | 13/33 (39.4) | 10/48 (20.8) | 2.28 (0.75–7.17) | 0.16 |
|  | 2 | 17/81 (21.0) | 8/33 (24.2) | 9/48 (18.8) | 1.31 (0.37–4.53) | 0.84 |
|  | 3 | 15/81 (18.5) | 8/33 (24.2) | 7/48 (14.6) | 1.70 (0.46–6.42) | 0.53 |
|  | 4 | 13/81 (16.0) | 6/33 (18.2) | 7/48 (14.6) | 1.18 (0.28–4.80) | 1.00 |
|  | 5 | 12/81 (14.8) | 5/33 (15.2) | 7/48 (14.6) | 0.97 (0.22–4.05) | 1.00 |
|  | 6 | 11/81 (13.6) | 5/33 (15.2) | 6/48 (12.5) | 1.18 (0.25–5.26) | 1.00 |

DR=durable response; EAC=endpoint assessment committee; TOI=Trial Outcome Index.

A patient was considered evaluable for TOI improvement if baseline TOI was ≤108 – magnitude (since TOI ranges from 0 to 108) and had at least one postbaseline TOI. TOI from unscheduled visits were not included.

*Odds ratio of DR versus non-DR and *P* value were obtained by fitting an exact logistic regression model using DR status (yes versus no) as predictor and TOI improvement (yes versus no) as response, stratified by disease stage (stage IIIB/IIIC/IVM1a versus IVM1b/IVM1c) and line of therapy (first line versus second line or greater).

# Supplementary Figure 1. Talimogene laherparepvec Duration of Response (per Investigator). As of the final analysis 93 of 295 patients (31.5%) randomized to talimogene laherparepvec had an overall response per investigator assessment (complete response, n=50; partial response, n=43) and 57 patients (19.3%) had a durable response.

**Study Protocol**

The trial protocol and details of statistical analysis for OPTiM have been previously published as an accompaniment to the primary report from the study, and can be found online at the *Journal of Clinical Oncology* website: http://jco.ascopubs.org/content/33/25/2780/suppl/DC1
