# Supplementary figures and images for: Durable response rate as an endpoint in cancer immunotherapy: insights from oncolytic virus clinical trials
Source: J Immunother Cancer. 2017 Sep 19;5:72. doi: 10.1186/s40425-017-0276-8 (PMC5604502; doi:10.1186/s40425-017-0276-8)

Figure S1

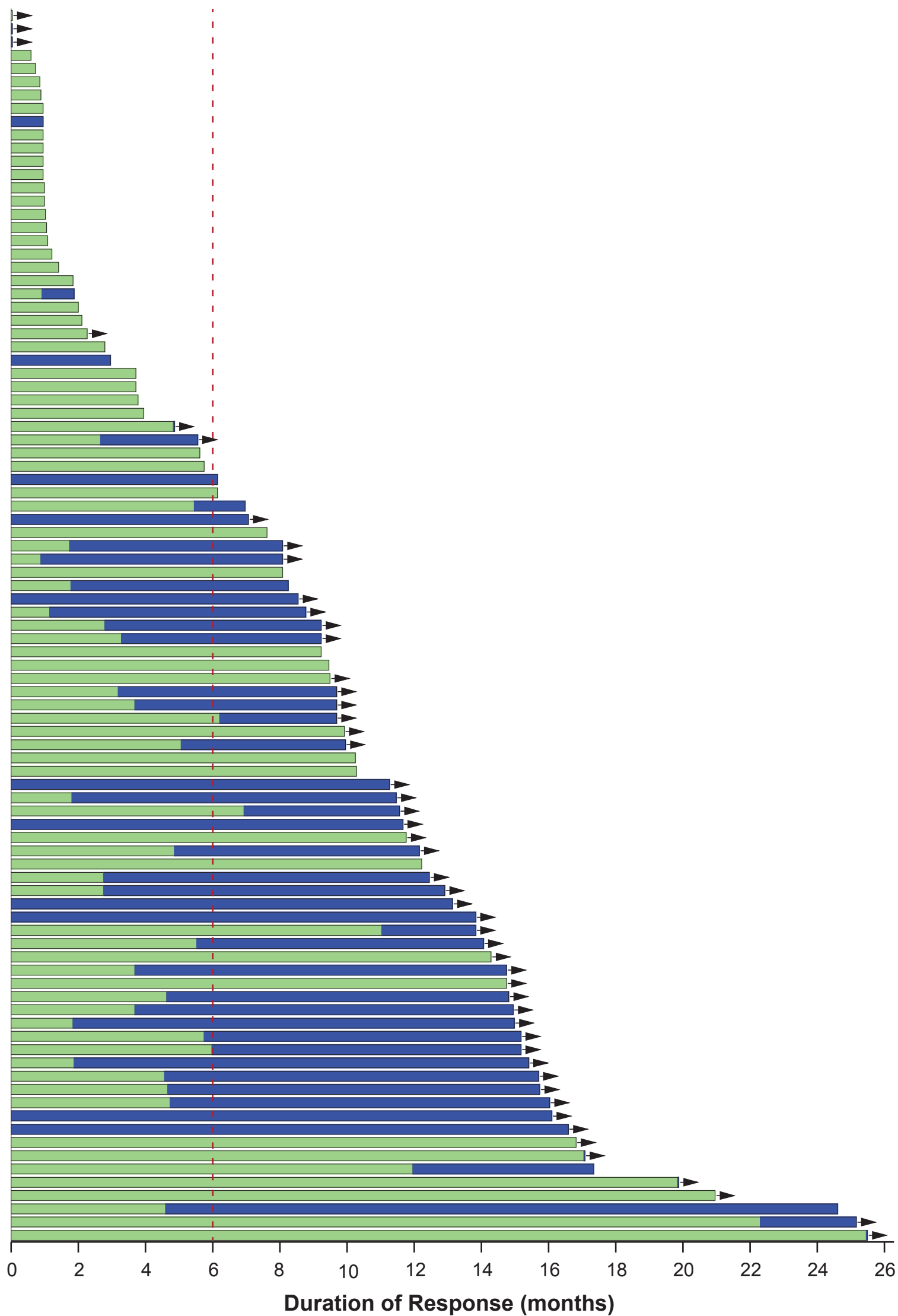

Supplement: Additional file 1: — Table S1. TOI Improvement Rate Association with Achievement of a DR (per EAC). Figure S1. Talimogene laherparepvec Duration of Response (per Investigator). As of the final analysis 93 of 295 patients (31.5%) randomized to talimogene laherparepvec had an overall response per investigator assessment (complete response, n = 50; partial response, n = 43) and 57 patients (19.3%) had a durable response. (ZIP 1510 kb) [file 40425_2017_276_MOESM1_ESM.zip › Figure S1.pdf]
